# Molecular response in the pectoral muscles and livers of broiler chickens to mitochondrial stimulation by *in ovo* administration of prebiotics

**DOI:** 10.2478/jvetres-2025-0061

**Published:** 2025-10-30

**Authors:** Aleksandra Dunisławska, Aleksandra Bełdowska, Olha Yatsenko, Jakub Biesek, Maria Siwek

**Affiliations:** Department of Animal Biotechnology and Genetics, Department of Animal Breeding and Nutrition, Bydgoszcz University of Science and Technology, 85-084 Bydgoszcz, Poland; Department of Animal Breeding and Nutrition, Bydgoszcz University of Science and Technology, 85-084 Bydgoszcz, Poland

**Keywords:** embryo stimulation, microbiome, mitochondria, MOS, XOS

## Abstract

**Introduction:**

Mitochondria are the primary sites for adenosine triphosphate production through oxidative phosphorylation, thus supporting the high metabolic demands of avian physiology. By administering prebiotics *in ovo*, the aim was to analyse how an early host-supporting strategy can modulate mitochondrial activity and affect the physicochemical composition of the pectoral muscles of chickens.

**Material and Methods:**

Three hundred incubated Ross 308 broiler eggs were injected: 60 with 0.2 mL of 0.2 mmol/L physiological saline (control group), and 60 each with 0.5 mg of xylotriose (XOS3 group), xylotetraose (XOS4 group), mannotriose (MOS3 group) or mannotetraose (MOS4 group) carried in 0.2 mL of physiological saline. On day 42 after hatching, the liver and pectoral muscle were collected from eight individuals from each group after sacrifice, and the muscle was evaluated physicochemically. Relative mitochondrial DNA (mtDNA) copy numbers were analysed in a real-time quantitative PCR (qPCR). Gene expression was determined by a reverse-transcription qPCR (RT-qPCR) for a mitochondrial gene panel.

**Results:**

The experimental factor was not shown to affect pectoral muscle weight. Water loss was significantly greater in the XOS4 group’s muscles. The overall mtDNA copy number was stable in both tissues. The XOS3 and MOS4 groups’ gene expression was significantly changed in pectoral muscle. Contrastingly, the XOS4 and MOS3 groups’ gene expression was more altered in the liver. Statistically significantly different expression was detected of the *CS, EPX, CYCS, TFAM* and *NRF1* genes in pectoral muscles and of all tested genes in livers.

**Conclusion:**

The potential of *in ovo* prebiotic administration is indicated as a strategic approach to optimise mitochondrial function, ultimately contributing to better growth rates and enhanced health in broiler chickens.

## Introduction

Mitochondria, referred to as the cell’s power plants, are key organelles responsible for the production of adenosine triphosphate (ATP) through oxidative phosphorylation. Their proper functioning is essential for maintaining cellular energy homeostasis, especially in broiler chickens characterised by exceptionally rapid growth rates and high metabolic requirements. Chicken breast muscle has a high mitochondrial content, which is essential for ensuring adequate aerobic metabolism and muscle efficiency. Not only the chicken’s skeletal muscle, but also its liver – as a central metabolic organ mediating nutrient processing from the gut – also relies heavily on mitochondrial function for its complex roles in detoxification, protein and lipid synthesis, and glucose metabolism. Supporting the mitochondria’s action and functioning is possible using appropriate nutritional strategies or supplementation. Selected ingredients such as omega-3 fatty acids, antioxidants such as vitamin C and zinc, and B vitamins can protect them against oxidative damage (7). Stimulation of the intestinal microbiota by bioactive substances should also be considered as one of the interventions for possible support of mitochondria in poultry. Products of microbiota metabolism, including short-chain fatty acids (SCFAs) such as butyrate, acetate and propionate, can influence mitochondrial function through several important mechanisms. These acids can activate G-protein–coupled receptors, leading to the regulation of signalling pathways such as adenosine monophosphate–activated protein kinase and mTOR pathways, which are crucial for maintaining energy balance and protein biosynthesis (28). Understanding the mechanisms regulating gut microbiota–mitochondria cross-talk is crucial for developing new therapeutic and preventive strategies. Dietary interventions such as prebiotic supplementation can modulate the gut microbiota in a manner that benefits mitochondrial function, translating into improved health and performance of broiler chickens.

Our previous analyses based on the same experimental system but performed on immune-related tissues showed that the total mitochondrial DNA (mtDNA) copy number is stable in both intestinal mucosa and caecal tonsils (8). A significant increase in gene expression in the caecal mucosa was induced by administration of xylotetraose and mannotriose. Administration of prebiotics to broilers caused downregulation of the expression of all analysed genes in caecal tonsil samples. These studies indicated that the effect of early stimulation of the gut microbiota on mitochondria in broiler chickens should be an important direction for further poultry intestinal health research. The extant relevant literature data focus primarily on the mutual interaction of the gut microbiota and mitochondria, especially in tissues related to metabolism and general homeostasis, *i.e*. liver and muscle tissue.

The aim of the study was to analyse how early dietary interventions (prebiotics delivered *in ovo*) can modulate mitochondrial activity in critical tissues such as the pectoral muscle and liver and also how they can affect the physicochemical composition of the pectoral muscles of broiler chickens.

## Material and Methods

### Experimental setup

The experimental design was described by Dunisławska *et al*. (8). The hatching eggs of a reproductive flock of Ross 308 broiler chickens were bought, and 300 were placed in an automated incubator (Jarson, Gostyń, Poland) at 37.8°C and in relative humidity of 61–63%. On day 12 of incubation, the eggs were randomly divided into five experimental groups (60 eggs per group). The groups were injected with one of (1) 0.2 mmol/L physiological saline (0.9%), creating the mock-injected control group; (2) a 0.5 mg dose of xylotriose, as the XOS3 group; (3) a 0.5 mg dose of xylotetraose, as the XOS4 group; (4) a 0.5 mg dose of mannotriose, as the MOS3 group; or (5) a 0.5 mg dose of mannotetraose, as the MOS4 group. A 0.2 mL volume of physiological saline solution of each substance was injected into the egg’s air chamber. On day 18, the eggs were moved to the hatcher (Jarson) and kept at 37.5°C and in relative humidity of 70–75%. On day 21, the chicks hatched. The birds enrolled were chosen for their body confirmation indices and weight.

Each group was divided into four replicates of 12 birds. The chickens were kept at a stocking density not exceeding 33 kg of livestock per 1 m^2^ of surface, in pens made from galvanised steel mesh. The floor was covered with wheat straw bedding. Environmental conditions were provided for broiler chickens as described by Biesek *et al*. (3). Three commercial diets were provided as appropriate for the stages of rearing: a starter diet was given from day 1 to day 14, a grower diet from day 15 to day 35 and a finisher diet from day 36 to day 42. The commercial diets met the nutritional requirements of broiler chickens as per recommendations (23). On day 42 of life, eight randomly selected individuals from each group were sacrificed. These birds’ livers and right pectoral muscles were collected and stored in a stabilising buffer (fixRNA; EURx, Gdansk, Poland) for future isolation of nucleic acids.

### Pectoral muscle physicochemical analysis

The pectoral muscles were weighed after 24 h of refrigerated storage (Hendi, Poznań, Poland) at 4°C. The pH was measured using a pH meter (Elmetron, Zabrze, Poland) with a dagger electrode. Buffers at pH 4.00, 7.00 and 9.00 were used for the calibration, as were selected previously by Biesek *et al*. (3). The colour was measured using a CR400 colorimeter (Konica Minolta, Tokyo, Japan) in the International Commission on Illumination (CIE) CIELAB scale, where L* is lightness, a* is redness and b* is yellowness, also adopting the protocol of Biesek *et al*. (3). The muscle’s ability to maintain water was analysed using the drip loss and water-holding capacity (WHC) methods. Drip loss determination consisted of weighing the pectoral muscle, making cuts in it, placing it in a small string bag and placing this bag in a larger imperforate one. After 24 h of storage of the samples prepared in this way at 4°C, the muscles were re-weighed. The percentage of water loss was calculated (14). The WHC method was performed according to Grau and Hamm (12). The muscles were minced in a meat grinder (Hendi), and a sample of 0.300 g (±0.005 g) was weighed and placed between two pieces of Whatman paper under a load of 2 kg for 5 min. The sample was reweighed, and the percentage of water loss was calculated.

### Analysis of mitochondrial gene expression

The method of gene expression analysis was described in Dunislawska *et al*. (8). Total RNA was isolated from liver and pectoral muscle tissue, which were processed in RNA Extracol (EURx) using a TissueRuptor homogeniser (Qiagen, Hilden, Germany) (n = 8/group). Ribonucleic acid was purified from the solution and contaminant using a GeneMATRIX Universal RNA Purification Kit (EURx) following the manufacturer’s instructions. The isolated RNA was subjected to quantitative and qualitative analysis using a NanoDrop 2000 UV-Vis spectrophotometer (Thermo Fisher Scientific, Wilmington, DE, USA) and electrophoresis on agarose gel. Isolated RNA was stored at –20°C. Gene expression analysis of the livers and pectoral muscles was performed by quantitative PCR with initial reverse transcription (RT-qPCR) against a panel of genes related to mitochondria: *CS* (citrate synthase), *EPX (MPO)* (eosinophil peroxidase), *CYCS* (cytochrome c), *TFAM* (transcription factor a, mitochondrial), *NRF1* (nuclear respiratory factor 1), *ND2* (nicotinamide adenine dinucleotide (reduced) dehydrogenase subunit 2 and *MnSOD (SOD2)* (superoxide dismutase 2, mitochondrial). Complimentary DNA (cDNA) was synthesised using the smART First Strand cDNA Synthesis kit (EURx). The qPCR reaction was performed using a LightCycler 480 Instrument II (RocheDiagnostics, Basel, Switzerland) as described in Dunisławska *et al*. (8). The qPCR reaction mixture contained SG (SYBR Green) onTaq qPCR Master Mix (EURx), 1μM of each primer (synthesised by Merck, Darmstadt, Germany) and 140 ng of cDNA. The sequences of primers were as designed for the previous study by the present authors (8) and are shown in Table 1. Relative gene expression analysis was conducted by applying the ΔΔCt method using *ACTB* (actin β) (22) as the reference gene (18). Statistical analysis was performed using Student’s *t*-test.

**Table 1.** Primer sequences used in the quantitative PCR with initial reverse transcription to analyse mitochondrial gene expression in broilers given early host-supporting prebiotic therapy

Gene	Full name	NCBI gene ID	Primer sequence (5ʹ→3ʹ)
*CS*	Citrate synthase	100858903	F: GCATTTTCCAAGGGTGAGCC R: CTGTAGGGCTGCAGGAGTG
*EPX (MPO)*	Eosinophil peroxidase	417467	F: AAGCAACTTCTGCAGGACTGA R: AGGTTGAGATGACCGCTCTG
*CYCS*	Cytochrome c	420624	F: CCATGAAGGTTGGGTCCAGT R: CTCTGTGTGTGTTCCTGGTCT
*TFAM*	Transcription factor a, mitochondrial	373888	F: CCTACGAGAGGGGAGGGG R: TCGATCCCTGTGTGAACTGC
*NRF1*	Nuclear respiratory factor 1	416677	F: AAAAGCCCAGAGCTGAATGGT R: GGCACCGTGCAAAGAGAGAA
*ND2*	NADH dehydrogenase subunit 2	63549482	F: ATCAGCCCTAATCCTCTTCTC R: GTGGCTATTGGGGTTATTTCT
*MnSOD (SOD2)*	Superoxide dismutase 2, mitochondrial	374042	F: GCAGCCTGTGCAAATCAAGA R: ACATCTCATCCATTTGCCTCTGA

1 NCBI – National Center for Biotechnology Information; F – forward; R – reverse; NADH – nicotinamide adenine dinucleotide (reduced)

### Relative mitochondrial DNA copy number

Total DNA was isolated from pectoral muscle and liver tissue using the GeneMATRIX Tissues DNA Purification Kit (EURx) according to the manufacturer’s instructions (n = 8/group). The isolated DNA was analysed quantitatively and qualitatively and then stored at –20°C.

### Relative mitochondrial DNA copy number analysis

Both matrices’ relative mitochondrial DNA copy numbers were determined using two different methods described in Dunisławska *et al*. (8). The molecular verification was provided using the LightCycler 480 Instrument II. As previously, the qPCR reaction mixture contained SG onTaq qPCR Master Mix intercalating dye (EURx), 1μM of each primer (also synthesised by Merck) and 2 μL of the sample (50 ng of DNA). The primers for the analysed genes, these being *D-loop* (displacement loop), *ATP6* (adenosine triphosphate synthase subunit 6), *ND6* (nicotinamide adenine dinucleotide (reduced) dehydrogenase subunit 6) and *GCG* (glucagon), were based on designs in the literature (35).

The mtDNA copy number was determined using the method of Zhang *et al*. (35). The average was calculated of the generated mtDNA copy numbers for individual genes. Student’s *t*-test was also administered to discover if differences between the control group and the individual experimental groups were statistically significant. Relative mtDNA copy number analysis was performed according to Venegas and Halberg (29). The *GCG* gene was used as the genomic DNA reference, and the *D-loop* gene as the mtDNA reference. Student’s *t*-test assessed differences between the control group and the individual prebiotic groups.

## Results

### Pectoral muscle physicochemical analysis

The physicochemical characteristics of the sampled pectoral muscles are presented in Table 2. The weight of pectoral muscles ranged from 471.96 to 533.40 g and was not affected by the experimental factor (P-value = 0.170). Also, all groups’ tissue had similar pH 24 h from slaughter, and their colour and drip loss were only insignificantly different (P-value > 0.05). However, statistically significantly higher water loss, expressed by WHC, was noticed in group XOS4 compared to other groups (P-value = 0.004).

**Table 2.** Physicochemical features of the pectoral muscles of broilers given early host-supporting prebiotic therapy

		Group					SEM	P-value
		Control	XOS3	XOS4	MOS3	MOS4		
Pectoral muscle (g)		533.40	505.04	482.34	483.70	471.96	8.660	0.170
pH_24hours_		5.94	6.06	6.05	6.03	6.03	0.010	0.119
Colour	L*	50.48	51.08	50.02	49.38	50.33	0,320	0.578
	a*	2.88	2.79	3.02	2.56	3.00	0,170	0.923
	b*	5.38	4.44	4.61	4.12	5.27	0,200	0.203
Drip loss (%)		1.35	1.56	1.34	1.28	1.65	0.060	0.191
Water-holding capacity (%)		30.39^b^	32.40^b^	39.55^a^	32.41^b^	34.70^ab^	0.840	0.004

1 XOS3 – xylotriose; XOS4 – xylotetraose; MOS3 – mannotriose; MOS4 – mannotetraose;

L* – lightness;

a* – redness;

b* – yellowness; WHC – water-holding capacity; SEM – standard error of the mean;

a,b – mean values marked with different letters in the row differ statistically significantly, P-value < 0.05

### Relative mitochondrial DNA copy number analysis

**Fig. 1.**
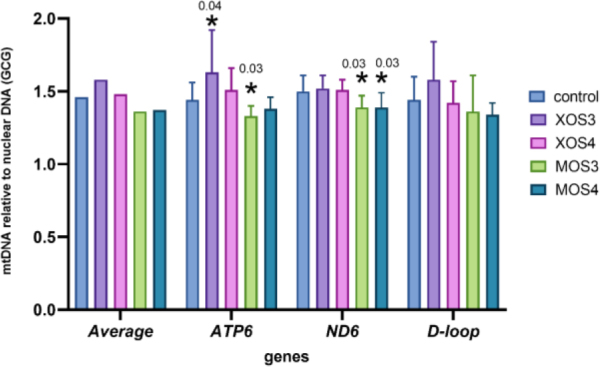
Mitochondrial DNA (mtDNA) copy number of adenosine triphosphate subunit 6 (*ATP6*), nicotinamide adenine dinucleotide (reduced) dehydrogenase subunit 6 (*ND6*) and displacement loop (*D-loop*) relative to nuclear DNA (glucagon gene – *GCG*) in the pectoral muscles of broiler chickens after *in ovo* stimulation with prebiotics. XOS3 – xylotriose; XOS4 – xylotetraose; MOS3 – mannotriose; MOS4 – mannotetraose; * – P-value ≤ 0.05

**Fig. 2.**
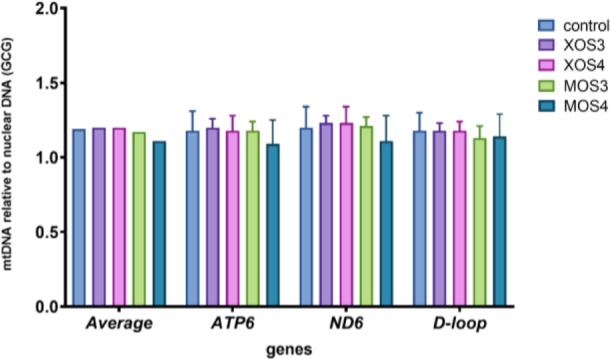
Mitochondrial DNA (mtDNA) copy number of adenosine triphosphate subunit 6 (*ATP6*), nicotinamide adenine dinucleotide (reduced) dehydrogenase subunit 6 (*ND6*) and displacement loop (*D-loop*) relative to nuclear DNA (glucagon gene – *GCG*) in the livers of broiler chickens after *in ovo* stimulation with prebiotics. XOS3 – xylotriose; XOS4 – xylotetraose; MOS3 – mannotriose; MOS4 – mannotetraose; * – P-value ≤ 0.05

**Fig. 3.**
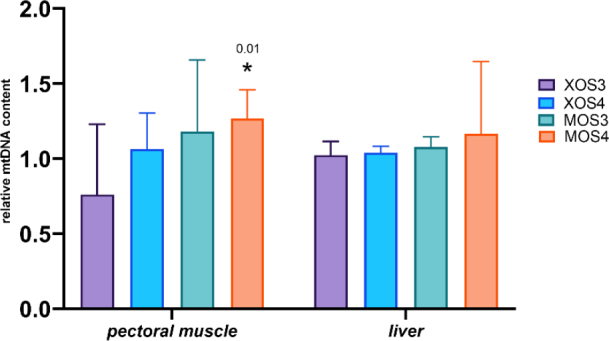
Relative mtDNA content in the pectoral muscles and livers of broiler chickens after *in ovo* stimulation with prebiotics. The *GCG* gene was used as the genomic DNA reference, and the *D-loop* gene as the mtDNA reference. XOS3 – xylotriose; XOS4 – xylotetraose; MOS3 – mannotriose; MOS4 – mannotetraose; * – P-value ≤ 0.05

The mitochondrial DNA copy numbers for the pectoral muscle samples are presented in Fig. 1, and those for the liver samples in Fig. 2. The pectoral muscles’ mean mitochondrial DNA copy number (mtDNA-CN) was 1.46, and was slightly higher in the XOS3 group at 1.58. The mtDNA-CN for the pectoral muscle *ATP6* gene in the XOS3 group significantly exceeded that of the control group at 1.63 *vs* 1.44, while the mtDNA-CN for this gene in this tissue in the MOS3 group was lower than the control copy number at 1.33. In the case of pectoral muscle *ND6*, a significantly lower mean mtDNA-CN resulted for the MOS3 and MOS4 groups, which was 1.39 for both. In the livers, the mtDNA-CNs of all genes in all groups were relatively similar. The relative mt DNA-CNs for the pectoral muscles and for the livers are presented in Fig. 3. The highest relative mt DNA-CN value for the pectoral muscle was detected in the MOS4 group, and the lowest in the XOS3 group. The values calculated for all experimental groups in the liver are close to 1.

### Mitochondrial gene expression

**Fig. 4.**
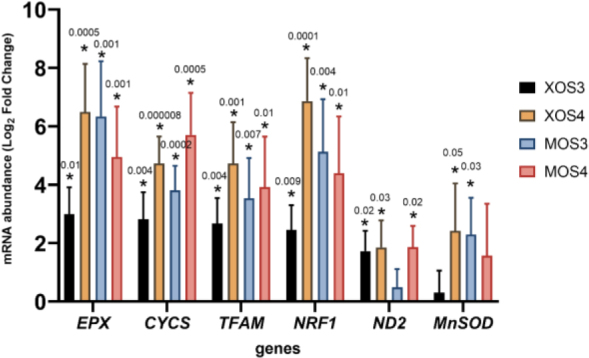
Expression of genes related to mitochondria in the livers of broiler chickens after *in ovo* stimulation with prebiotics. XOS3 – xylotriose; XOS4 – xylotetraose; MOS3 – mannotriose; MOS4 – mannotetraose; * – P-value ≤ 0.05

**Fig. 5.**
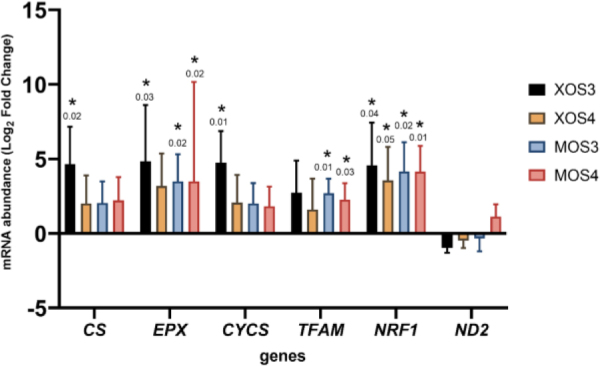
Expression of genes related to mitochondria in the pectoral muscles of broiler chickens after *in ovo* stimulation with prebiotics. XOS3 – xylotriose; XOS4 – xylotetraose; MOS3 – mannotriose; MOS4 – mannotetraose; * – P-value ≤ 0.05

Gene expression results in the pectoral muscles are presented in Fig. 4. Statistically significant results were detected for six of the seven genes analysed (*EPX, CYCS, TFAM, NRF1, ND2* and *MnSOD*). Both prebiotics upregulated the expression of these genes compared to the expression in control chicken tissue. The greatest increase in expression occurred in the XOS3 group. Gene expression results in the livers are shown in Fig. 5. All four prebiotics upregulated the entire set of analysed genes. Statistically significantly higher gene expression results were detected for all seven analysed genes compared to expression in liver tissue from chickens given no *in ovo* prebiotics.

## Discussion

Mitochondria and bacterial gut microbiota have some features in common. They share a prokaryotic origin, with mitochondria having evolved from the *Alphaproteobacteria* bacterial phylum or an ancestor related to *Rickettsiales* (2). Both mitochondria and gut microbiota possess circular genomes and prokaryotic ribosomes which are passed down maternally (20), and which are susceptible to aminoglycosides, quinolones and beta-lactams known to set up mitochondrial dysfunction. Nutrient metabolism is yet another function shared by mitochondria and gut microbiota. The gut microbiome produces metabolites that influence mitochondrial function and replication within a cell, which increases ATP production (10). The cross-talk between gut microbiota and mitochondria might be modulated by SCFAs, especially butyrate, and by bile acids and direct modulation of gene expression (2). In the current experiment, prebiotics administered *in ovo* did not affect SCFAs, but in previous research we did find an increased level of bile acids in the caecum of broiler chickens that received MOS4 *in ovo* (1).

Bile reaches the caecum after being transported into the chicken small intestine through the biliary system, which begins in the liver. This organ is also the first one that is exposed to the gut system and that receives gut microbiota components and metabolites present in the blood. Therefore, liver homeostasis is derived from the beneficial effect of commensal gut microbiota, and a dysbiosis in gut microbiota (potentially induced through altered mitochondrial signalling) might cause liver damage (33).

Mitochondrial activity plays a central role in maintaining metabolic homeostasis not only in the liver, but also in muscle tissue, these two organs both being deeply influenced by gut-derived signals and nutritional interventions. The relation between metabolic health and mitochondrial health is particularly important in the case of muscle. Muscles have the highest mitochondrial density and are the most metabolically active tissues. Mitochondria and muscle function are influenced by diet (10). Probiotic supplementation with *Lactobacillus plantarum* has a particularly strong effect on muscle performance and antioxidative capacity, the latter being related to meat quality. There is a series of metabolic processes that occur after slaughter which have an impact on meat quality. Residual muscle energy metabolism immediately after slaughter initiates the biochemical processes – such as pH decline and protein breakdown – that govern the transformation of muscle into meat. The key organelle of cellular metabolism is the mitochondrion, and since mitochondria remain intact and functional during first hours after slaughter, they can impact post-mortem muscle metabolism and affect meat quality (31).

### Pectoral muscle physicochemical features

The bioactive substance given in *in ovo* injection during broilers’ embryogenesis primarily ameliorates their health status. Additional highly important gains from this host-supporting strategy are in the birds’ performance and the quality of the meat. *In ovo* administration of a synbiotic on day 12 of incubation at 2 and 3 mg/embryo doses suspended in 0.2 mL of physiological saline had no effect on the pH or colour of broiler pectoral muscles, as Tavaniello *et al*. (26) reported. These results correspond to those of our research, and the values of pH and colour were similar. Another study by Tavaniello *et al*. (27) used two different synbiotics, *Lactobacillus salivarius* + galactooligosaccharides or *Lactobacillus* + raffinose family oligosaccharides, in Cobb broiler chickens. This *in vivo* research also showed no effect on pectoral muscles’ pH, colour or WHC 24 h from sample collection. Similarly, Maiorano *et al*. (19) and Cheng *et al*. (5) concluded that synbiotic supplementation in broilers had no adverse effect on meat quality parameters.

Optimal muscle pH and ability to maintain water are desirable for the mechanised processing of meat (17). In our research, the injection of prebiotics had no adverse effect on these features, which is beneficial for further processing in the poultry meat industry and, along with unaffected colour, for meat reception in the consumer market. Lee *et al*. (17) showed that broiler meat with L* < 56 is dark meat. In our research, the L* value was approximately 50. However, the cited authors measured the colour on the bone side of the muscle, in contrast to our method, where the outer part of the muscle was taken. The WHC was higher in the group administered xylotetraose than in the others (P > 0.05), which is adverse. The ability to maintain water in muscle fibres affects tenderness, chewiness and processing under high temperatures. As Bowker and Zhuan (4) described, WHC is a complex trait related to structural and biochemical changes during the transformation of muscle tissue post slaughter. Higher WHC is mainly an improvement striven for in countering pale, soft, exudative (PSE) meat, which is a broiler meat problem. The changes condemning meat as PSE are primarily influenced by post-mortem metabolism, pH and protein denaturation. Tavaniello *et al*. (27) noticed no effect on the WHC after *in ovo* synbiotic administration. However, it should be noted that results may depend on individual birds’ muscle structures and their chemical composition, including their fat, protein and water content. The changes in the structure of muscle tissue after mincing can also heighten the differences in water loss from meat (15). Zhang *et al*. (32) reported that L-ascorbic acid injection during incubation affected *in vivo* results in reducing the shear force of the pectoral muscle, which corresponded to findings made by Ferreira *et al*. (9). These results suggest an improving effect of *in ovo* administration on broiler meat quality.

### Relative mitochondrial DNA copy number analysis

Mitochondria contain their own genetic material, mtDNA, which plays an important role in metabolism, apoptosis and intracellular communication. The mtDNA copy number reflects the DNA’s function and oxidant-induced cell damage, and the parameter varies with age, tissue and sex. In broiler chickens, the mtDNA copy number was related to the ascites phenotype in a tissue-specific manner. Zhang *et al*. (35) performed a complex analysis of variation in mtDNA copy number based on the mitochondrial *D-loop, ND6* and *ATP6* genes in various tissues of 21-day-old broiler chickens. The highest number of mtDNA copies was determined in brain cells, and blood had the lowest number. The numbers of mtDNA copies in the livers and pectoral muscles had intermediate values. Similar results were obtained in the current study for mtDNA copy number in broiler chickens’ liver and pectoral muscle tissue from birds stimulated *in ovo* with xylooligosaccharides and mannooligosaccharides. Overall, the mtDNA copy number was numerically higher in the livers than in the muscles regardless of the prebiotic used for *in ovo* stimulation. The only statistically significant difference in the numbers for each tissue was noted in the pectoral muscle of broiler chickens from the MOS4 group. This muscle is where industrial chicken farming has influenced mtDNA copy numbers: the intensive selection of broiler chickens for better feed conversion efficiency and muscle weight has significantly affected the mitochondrial content of the pectoralis major muscle. In 1977, the mitochondrial content in the pectoral muscles of broiler chickens was estimated at 4.1%, while 45 years later it had halved as a result of selective breeding (24). The mtDNA copy number, its maintenance and mitochondrial gene expression are controlled by the nuclear encoded factors. The most significant role in this process is played by *TFAM* (13). In the current study, the expression of the *TFAM* gene was upregulated in the pectoral muscles of chickens that received MOS4 *in ovo*.

### Mitochondrial gene expression

The activity of mitochondria after embryonic stimulation of the host microbiota with XOS and MOS was estimated based on a panel of genes belonging to markers of mitochondrial biogenesis (*NRF1* and *TFAM*), oxidative metabolism (*CS*), mitochondrial superoxide generation (*MnSOD*), electron transport (*CYCS*), inflammatory response (*MPO*) and mitochondrial reactive oxygen species production (*ND2*). The same panel was used to evaluate mitochondrial activity in caecal mucosa and tonsils (8).

The *NRF1* gene encodes a protein responsible for mitochondrial DNA transcription and replication. The *TFAM* gene is a key transcription factor in mitochondria. The protein encoded by this gene is responsible for mitochondrial DNA replication and repair. The *CS* gene encodes a protein that catalyses citrate synthesis from oxaloacetate and acetyl coenzyme A. This gene is also a biomarker of mitochondrial content. The *MnSOD* gene encodes a mitochondrial protein, forming a homotetramer and binding one manganese ion per subunit. A haem protein that functions as a central component of the electron transport chain in mitochondria is what is encoded by the *CYCS* gene. Its activity is necessary for life. However, the protein encoded by *CYCS* also initiates apoptosis. Eosinophil peroxidase is involved in the innate immune system. An important paralogue of this gene is *EPX* (25). The *ND2* gene is a subunit of NADH dehydrogenase, and this gene is involved in the production of mitochondrial reactive oxygen species (34).

The administration of xylotriose, xylotetraose, mannotriose and mannotetraose prebiotics *in ovo* resulted in enhanced oxidative metabolism through mitochondria biogenesis. The effect was slightly more pronounced in the liver, where the upregulation of *NRF* and *TFAM* was detected in all experimental groups. Meanwhile, in the pectoral muscle, the upregulation of both genes (NRF and *TFAM*) was shown uniquely in the MOS groups. Similarly, the beneficial effect of bioactive substances on mitochondrial biogenesis was presented by Kikusato *et al*. (16) in an *in-vitro* model of avian cell muscle supplemented with oleuropein. Another outcome of the study by Kikusato *et al*. (16) consistent with the current report is the suppression of mitochondrial superoxide generation due to the upregulation of *MnSOD* gene expression. This positive effect was also detected in the livers of broiler chickens from the XOS4 and MOS3 groups.

Statistically significant modulation of *ND2* gene expression was detected in liver tissue only. Upregulation of *ND2* expression was detected in three experimental groups: XOS3, XOS4 and MOS4. The expression of *ND2* in liver tissue might be related to this organ’s energy requirement (34). As presented in the current study, the prebiotic administration during early embryogenesis significantly changed hepatocytes which might have boosted the energy supply to support cellular function in the organs and tissue of 42-day-old broiler chickens.

Cytochrome c is known for its role in ATP synthesis in mitochondria. However, upon an apoptotic stimulus, cytochrome c is released into the cytosol to initiate programmed cell death (21). Upregulation of *CYCS* gene expression and increased expression of the *NRF* and *TFAM* genes might suggest higher mitochondrial biogenesis in the livers of all experimental groups. Mitochondrial biogenesis is beneficial for the host organism and might be applied as a therapeutic strategy for treating acute and chronic mitochondrial diseases (30). We suggest that xylooligosaccharides (XOS3 and XOS4) and mannooligosaccharides (MOS3 and MOS4) might be recognised as potential indirect activators of mitochondrial biogenesis.

According to a study by Choi *et al*. (6), EPX is involved in several immunological functions such as bactericidal activity against a wide range of microorganisms or eosinophil extracellular trap formation. When expressed in the liver, *EPX* is believed to be involved in tissue homeostasis and repair (11). It might therefore be speculated that the upregulation of *EPX* gene expression by xylooligosaccharides and mannooligosaccharides administered *in ovo* benefits the livers of fast-growing broiler chickens.

## Conclusion

These findings highlight the potential of administering prebiotics *in ovo* on day 12 of egg incubation as an effective strategy to enhance mitochondrial function, which can lead to improved growth rates and overall health in broiler chickens. This research not only deepens our understanding of mitochondrial dynamics in birds, but also paves the way for innovative nutritional approaches designed to boost poultry production efficiency by specifically targeting mitochondrial activity.
